# Halo: a pretrained model for whole-cell segmentation from nuclei images in spatial transcriptomics

**DOI:** 10.1093/bib/bbag515

**Published:** 2026-09-17

**Authors:** Xingyuan Zhang, Haotian Zhuang, Zhicheng Ji

**Affiliations:** Department of Biostatistics and Bioinformatics, Duke University School of Medicine, 2424 Erwin Road, Durham, NC, 27705, United States; Program of Computational Biology and Bioinformatics, Duke University School of Medicine, 2424 Erwin Road, Durham, NC, 27705, United States; Department of Biostatistics and Bioinformatics, Duke University School of Medicine, 2424 Erwin Road, Durham, NC, 27705, United States; Department of Biostatistics and Bioinformatics, Duke University School of Medicine, 2424 Erwin Road, Durham, NC, 27705, United States; Program of Computational Biology and Bioinformatics, Duke University School of Medicine, 2424 Erwin Road, Durham, NC, 27705, United States

**Keywords:** spatial transcriptomics, cell segmentation, imaging

## Abstract

Spatial transcriptomics (ST) enables measurement of gene expression while preserving spatial organization within tissues. Accurate reconstruction of single-cell transcriptomes requires precise whole-cell segmentation, yet many ST experiments provide only nuclear staining images, making reliable inference of cell boundaries difficult. Here we introduce Halo, a pretrained segmentation model that reconstructs whole-cell boundaries by integrating nuclear morphology with the spatial distribution of RNA transcripts. Halo converts transcript coordinates into molecular density maps that are processed jointly with DAPI images using a Cellpose-SAM segmentation architecture. Halo is pretrained on multimodal Xenium data from 12 tissue types and can be directly applied to new datasets without additional training, providing a ready-to-use alternative to supervised approaches that require dataset-specific fitting. Across diverse tissues, Halo achieves substantially higher agreement with the 10*×* multimodal reference segmentation than existing methods, in terms of both cell boundaries and RNA-to-cell assignments, while requiring only DAPI staining and transcript spatial information. Improved segmentation leads to more reliable cell-type identification and more accurate estimation of cell morphological features. By providing a pretrained, generalizable model for whole-cell reconstruction, Halo enables scalable and reproducible cell segmentation for image-based ST.

## Introduction

Spatial transcriptomics (ST) is a rapidly evolving technology that enables the measurement of gene expression while preserving the spatial organization of cells within tissues, providing critical insights into tissue architecture, cell–cell interactions, and spatially organized biological processes [1–4]. Broadly, ST platforms can be categorized into spot-based [5, 6] and image-based approaches [7, 8]. Spot-based methods profile transcripts captured within predefined spatial locations, or spots, each of which typically contains RNA from multiple neighboring cells, resulting in limited spatial resolution. In contrast, image-based methods detect transcripts directly within tissue sections using imaging and *in situ* hybridization or sequencing strategies, achieving single-cell or even subcellular resolution. Owing to this finer spatial granularity, image-based approaches enable more precise characterization of cellular heterogeneity and spatial gene expression patterns, and are particularly powerful for resolving complex tissue microenvironments.

In addition to providing spatial locations of RNA transcripts, image-based ST technologies also generate complementary fluorescence imaging data. For example, the widely used 10*×* Xenium platform offers two imaging configurations. The first includes DAPI staining for imaging cell nuclei, while the second, multimodal configuration additionally captures signals from cell boundaries, intracellular RNA, and other molecular markers alongside DAPI staining. This rich imaging information makes it possible to infer cell locations and boundaries through a computational procedure known as cell segmentation [9–12]. Cell segmentation is a critical step for assigning transcripts to individual cells and constructing single-cell gene expression matrices, which serve as the basis for most downstream analyses, including cell-type annotation [13, 14], spatial domain detection [15], and identification of spatially variable genes [16, 17].

**Figure 1:**
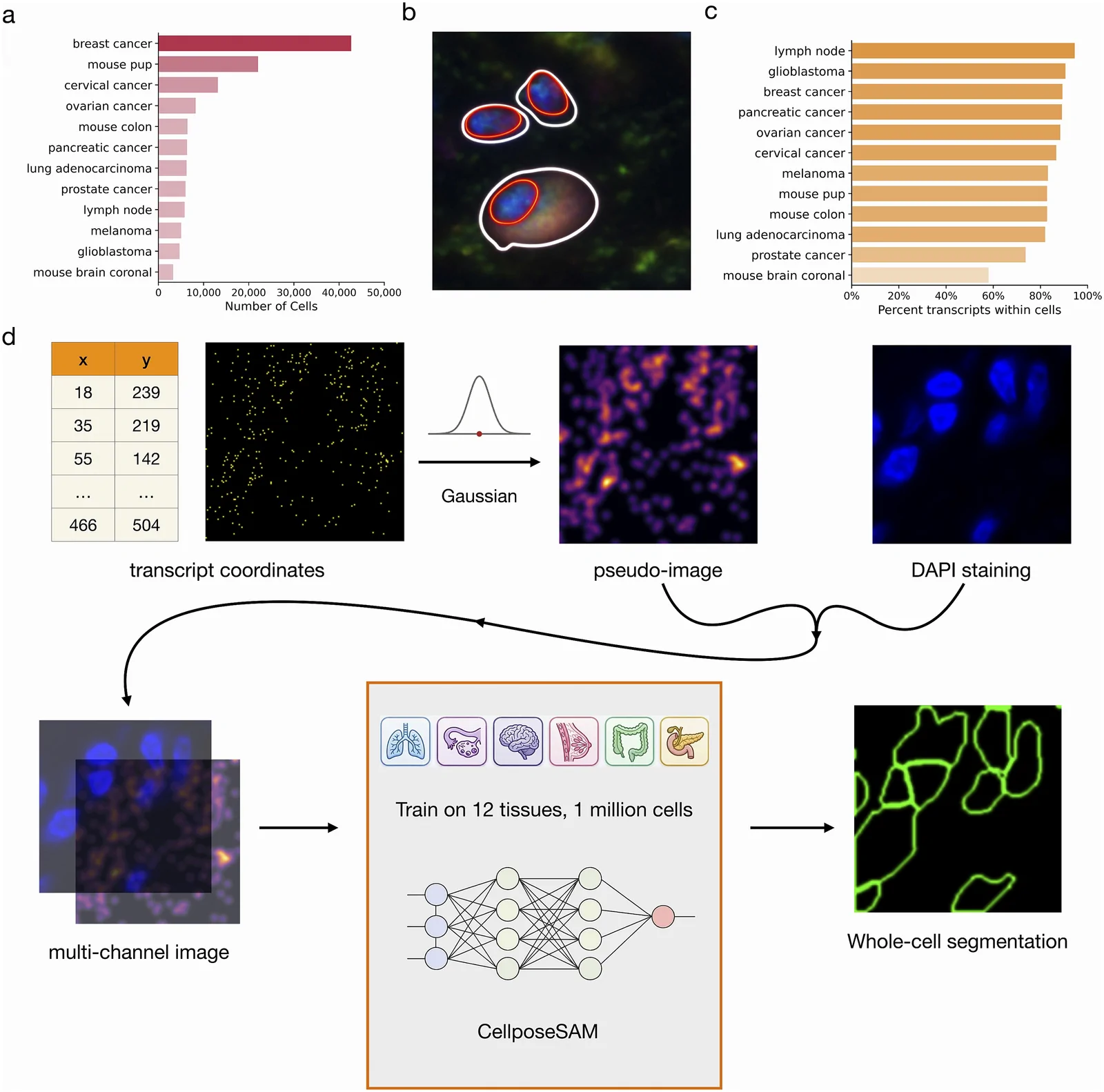
Overview of the motivation and workflow of Halo. (a) Number of cells segmented based on nuclei staining across Xenium datasets with multimodal imaging. (b) Whole-cell boundaries (white contours) and nuclear boundaries (red contours) for three representative cells. (c) Percentage of RNA transcripts within cell boundaries across Xenium datasets. (d) Overview of Halo.

Ideally, cell segmentation aims to reconstruct whole-cell boundaries rather than only nuclear boundaries, because transcripts located in the cytoplasm carry important information about cell identity [18]. However, accurate whole-cell segmentation remains challenging when only DAPI-stained images are available, as these images label only nuclei and provide little direct information about the surrounding cytoplasm or cell membrane. Even when multimodal imaging is available, which often requires substantially greater financial and experimental resources, tens of thousands of cells may still lack reliable signals from additional imaging channels and, therefore, rely primarily on nuclei staining to infer whole-cell boundaries (Fig. 1a). In such settings, existing image-based segmentation methods [9–11] can reliably delineate nuclei but generally cannot reconstruct accurate whole-cell boundaries.

A widely adopted strategy, implemented in the 10*×* Xenium Ranger analysis pipeline, addresses this limitation by first segmenting nuclei from DAPI images and then expanding each nuclear mask by a fixed number of pixels to approximate the corresponding whole-cell boundary. However, this approach has several major limitations (Fig. 1b). First, whole-cell morphology can differ substantially from nuclear morphology. Second, the distance between the nucleus and the cell boundary varies widely across cells. Third, the nucleus is not always centrally located within a cell. As a result, uniformly enlarging nuclei by a fixed number of pixels often fails to accurately capture true cell boundaries and can lead to inaccurate assignment of transcripts to cells.

Several recent computational methods have attempted to improve segmentation accuracy by integrating RNA spatial coordinates with imaging information [19, 20]. These approaches demonstrate that the spatial distribution of transcripts contains useful information for delineating cellular boundaries. However, existing methods typically require dataset-specific model training and annotated segmentation masks, which limits their applicability across datasets and tissue types. In practice, many ST datasets lack high-quality ground-truth segmentation masks, making it difficult to apply such approaches broadly.

To address this limitation, we developed Halo, a pretrained segmentation model that reconstructs whole-cell boundaries by integrating nuclear morphology with the spatial distribution of RNA transcripts. Halo leverages the observation that most transcripts reside within cells and, therefore, collectively encode information about cellular extent (Fig. 1c). To integrate transcript spatial information with nuclear imaging, Halo converts RNA coordinates into molecular density maps that can be processed jointly with DAPI images within a modern segmentation architecture. The model is trained on multimodal Xenium datasets spanning 12 tissue types, enabling it to learn generalizable relationships between nuclear morphology, transcript spatial patterns, and whole-cell structure. As a result, Halo can be directly applied to new ST datasets without requiring additional model training or annotated segmentation masks.

We demonstrate that Halo achieves substantially higher agreement with the 10*×* multimodal reference segmentation than the commonly used nuclear expansion (NE) strategy across diverse tissue types. By providing a pretrained and generalizable model for whole-cell reconstruction, Halo enables scalable and reproducible cell segmentation for image-based ST analyses.

## Results

### Halo overview

Halo predicts whole-cell boundaries by jointly leveraging RNA spatial coordinates and DAPI nuclear staining images. The name Halo reflects this design, as each DAPI-stained nucleus serves as a central anchor, while the surrounding RNA transcripts form a molecular “halo” that informs the inferred full-cell extent. To integrate these heterogeneous modalities, it first converts RNA spatial location data into pseudoimages, transforming molecular information into an image-compatible representation that can be processed alongside nuclei images within a unified framework. A Cellpose-SAM model [21], a recent foundation model for biological segmentation, is then trained on multi-channel inputs comprising the RNA pseudoimage and the DAPI image to infer whole-cell boundaries. Cellpose-SAM adapts the pretrained transformer backbone of the Segment Anything Model (SAM) [22] into the Cellpose framework, combining SAM-derived generalizable image representations with the Cellpose formulation for biological instance segmentation. Specifically, the SAM backbone provides a powerful image encoder that can extract transferable visual features from diverse image types, while the Cellpose framework predicts cell instances using its established representation of cell shape and spatial organization. By integrating these components, Cellpose-SAM provides a segmentation model that is more broadly generalizable across biological image modalities, object sizes, and staining patterns than models trained for a single narrowly defined setting. In Halo, this architecture allows us to use Cellpose-SAM as a flexible segmentation backbone and fine-tune it for whole-cell boundary inference from two complementary inputs, nuclear morphology captured by DAPI staining and transcript-derived molecular context captured by the RNA pseudoimage.

Halo is trained using multimodally stained samples generated on the 10*×* Xenium platform across 12 distinct tissue types (Fig. 1d), enabling robust performance and broad applicability across diverse tissue contexts. Halo can be directly applied to a new dataset using RNA spatial coordinates and DAPI nuclear staining images as inputs.

### Halo improves whole-cell segmentation and transcript assignment

**Figure 2:**
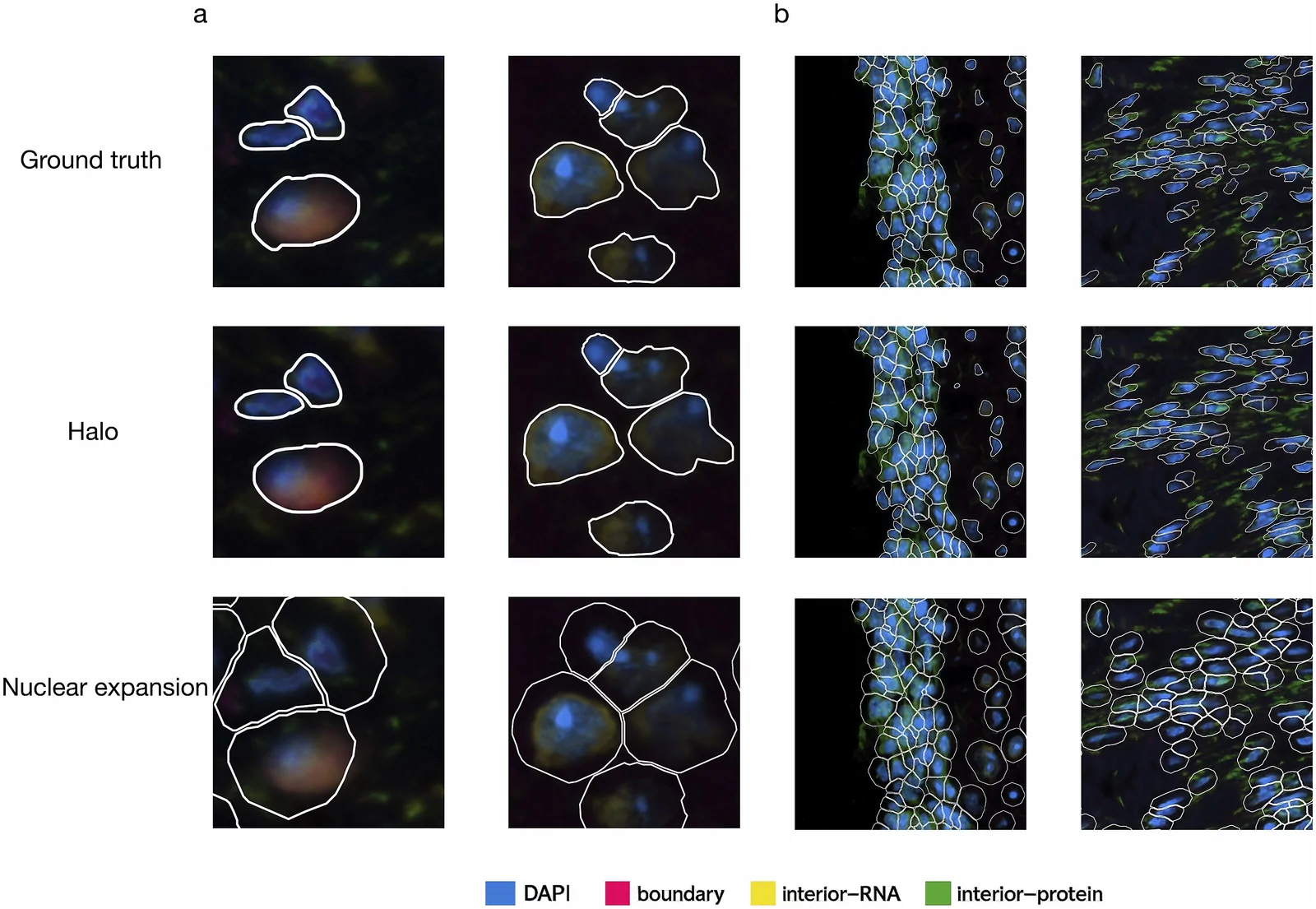
Whole-cell segmentations (white boundaries) of representative cells for ground truth, Halo, and NE. (a) Zoomed-in examples, left, human pancreatic cancer, right, mouse brain coronal. (b) Zoomed-out examples, left, human glioblastoma, right, human melanoma.

We applied Halo to image tiles in the test set that were not used during Halo’s training. As a competing approach, we also applied the NE method. Figure 2 compares the ground-truth whole-cell segmentation with the segmentation results obtained by Halo and NE in four representative regions from different tissues. The segmentation produced by Halo is highly consistent with the ground truth. In contrast, the NE results deviate from the ground truth in most cases, exhibiting two major issues. First, NE typically produces round or elliptical boundaries that fail to capture the more complex cell boundary contours observed in the ground truth. Second, the boundaries generated by NE are generally larger than those in the ground truth.

**Figure 3:**
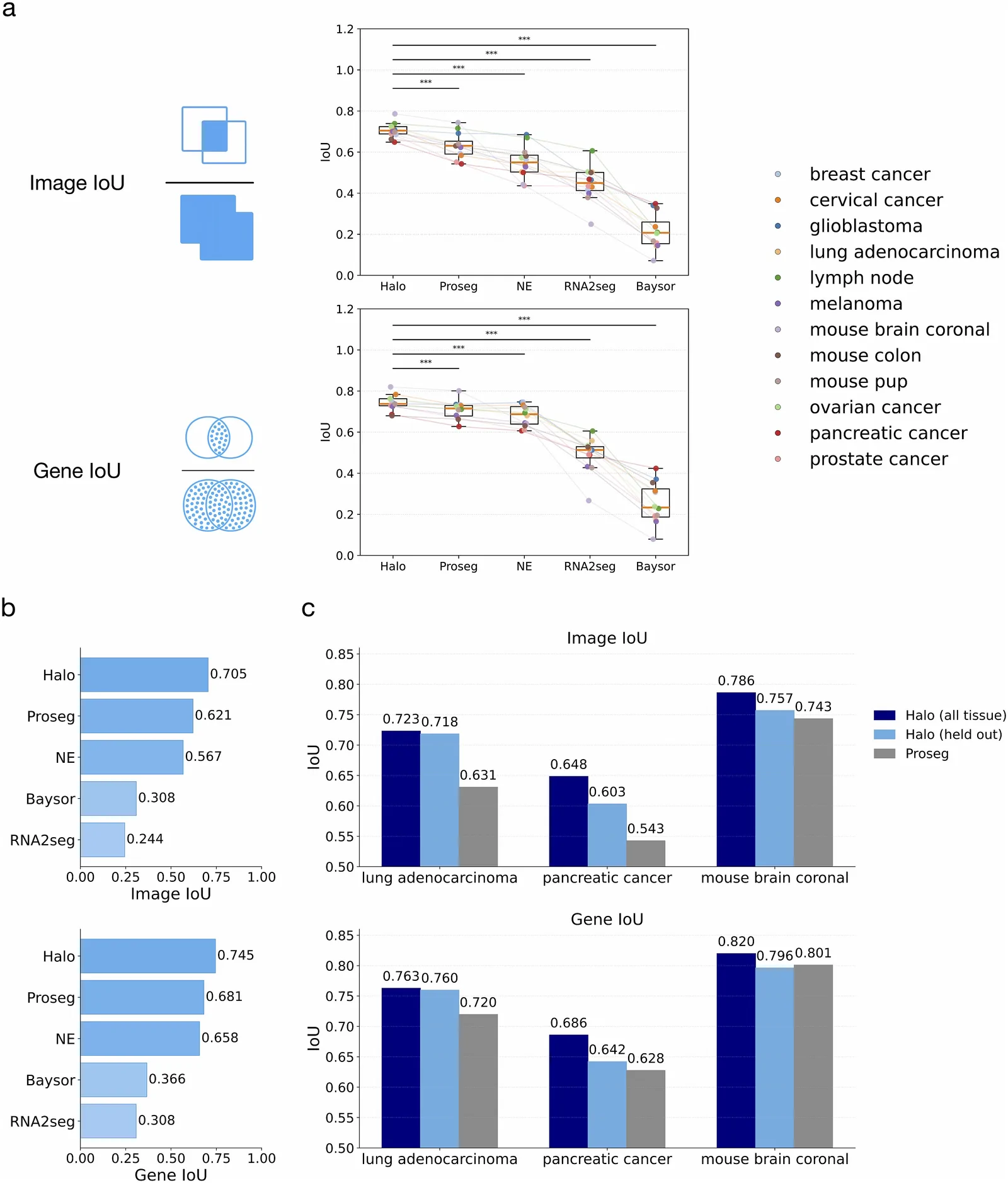
Evaluation of Image IoU and Gene IoU comparing Halo with competing methods. (a) Image IoU (top) and Gene IoU (bottom) across the 12 tissues used to train the full Halo model. Each point represents the dataset-level mean IoU across test tiles for that tissue. Statistical significance was assessed using a paired two-sided Wilcoxon signed-rank test. *** indicates *P*-value <.001. (b) Image IoU (top) and Gene IoU (bottom) for human renal cell carcinoma. No statistical test was performed because only one independent dataset was available. (c) Image IoU and Gene IoU for the three held-out tissues, comparing the full Halo model trained on all 12 tissues (all tissues), the Halo model trained on the remaining nine tissues (held out), and Proseg.

We then systematically evaluated the performance of Halo across different tissue types in a held-out-tile evaluation study, using image intersection over union (IoU) to quantify the agreement between whole-cell masks generated by Halo and the ground truth (Fig. 3). The training and test sets included the same tissue types. IoU is a standard metric for evaluating cell segmentation quality and has been widely used in previous studies [9–11]. We compared Halo with four competing methods: NE, Baysor [23], Proseg [24], and RNA2seg [20]. Baysor and Proseg are unsupervised probabilistic methods that infer cell boundaries from the spatial distribution of transcripts, whereas RNA2seg is a deep learning method that integrates transcript locations with nuclear images through a teacher–student training framework. Halo achieved the highest image IoU for every tissue type. Across all tissues, Halo attained a median image IoU of *∼*0.70, exceeding Proseg by *∼*0.10 and NE by *∼*0.15, with significantly higher Image IoU than all competing methods. These results indicate that the whole-cell boundaries reconstructed by Halo show higher agreement with the 10*×* reference segmentation than those produced by the competing approaches.

Once whole-cell boundaries are obtained, an RNA transcript can be assigned to a cell if its spatial location falls within the cell boundary. To quantitatively evaluate this step, we devised gene IoU, a metric analogous to the image IoU described above, to assess the agreement between RNA–cell assignments derived from Halo or competing methods and those from the ground truth (Fig. 3). Halo consistently achieves higher gene IoU values than the competing methods across nearly all tissue types. Across tissues, Halo attains a median gene IoU of nearly 0.75, compared with *∼*0.70 for Proseg. NE achieves a lower median gene IoU, while both Baysor and RNA2seg perform worse than NE. These results indicate that Halo shows higher agreement with the 10*×* reference segmentation than the alternative segmentation methods, with the largest overall improvement observed relative to Baysor and RNA2seg.

To evaluate Halo and the competing methods on a tissue type not represented in the training data, we applied all methods to an independent human renal cell carcinoma Xenium dataset representing an unseen tissue type and imaging sample. The dataset was released after Halo model training and was therefore unavailable during model development. It was generated using the same Xenium platform as the training data and additionally includes protein profiling. However, only the DAPI image and transcript spatial information were used for Halo evaluation, and the protein measurements were not used. Halo achieved an image IoU of 0.705 and a gene IoU of 0.745, outperforming all competing methods on both metrics (Fig. 3b). These results are comparable to Halo’s average performance in the preceding evaluations, providing evidence that Halo can generalize to previously unseen tissue and imaging contexts. Additionally, we performed a held-out-tissue analysis in which three tissues were completely excluded from Halo model training and used exclusively for testing, while the remaining nine tissues were used for training. Halo showed slightly lower performance than the model trained on all tissues, but on average still outperformed Proseg, the strongest competing method in our comparison, across the three held-out tissues (Fig. 3c). NE, Baysor, and Proseg do not require tissue-specific training, while RNA2seg was evaluated using the pretrained model released by the original study. Thus, their performance is unaffected by which tissues were held out from Halo training.

In addition to Image IoU and Gene IoU, we designed an incompatible-marker co-assignment score to provide a reference-independent assessment of biological plausibility. Because a single cell is unlikely to simultaneously contain markers characteristic of multiple distinct cell types, a lower score indicates less mixing of biologically incompatible marker transcripts. As shown in Supplementary Fig. S1, Halo exhibited incompatible-marker co-assignment scores comparable to those of the other segmentation methods, including NE. Thus, although Halo showed substantially higher agreement with the 10*×* multimodal reference segmentation according to Image IoU and Gene IoU, the reference-independent incompatible-marker metric did not clearly distinguish the methods.

Finally, we evaluated transcript assignment agreement with the 10*×* reference segmentation as a function of the distance between each transcript and its corresponding or nearest 10*×* cell centroid (Supplementary Fig. S2). Halo maintained consistently high assignment agreement across all distance intervals and showed the greatest improvement over the competing methods for transcripts located 5–10 *μ*m from the cell centroid.

### Halo improves cell clustering and cell-type identification

Once RNA transcripts are assigned to cells and a single-cell count matrix is generated, a key task is to perform cell clustering and identify the cell type of each cell cluster based on its transcriptomic profile. The accuracy of cell-type identification directly affects several downstream analyses that rely on cell-type information, such as cellular neighborhood and interaction analysis [25, 26], and the identification of cell-type-specific spatially variable genes [16, 27].

**Figure 4:**
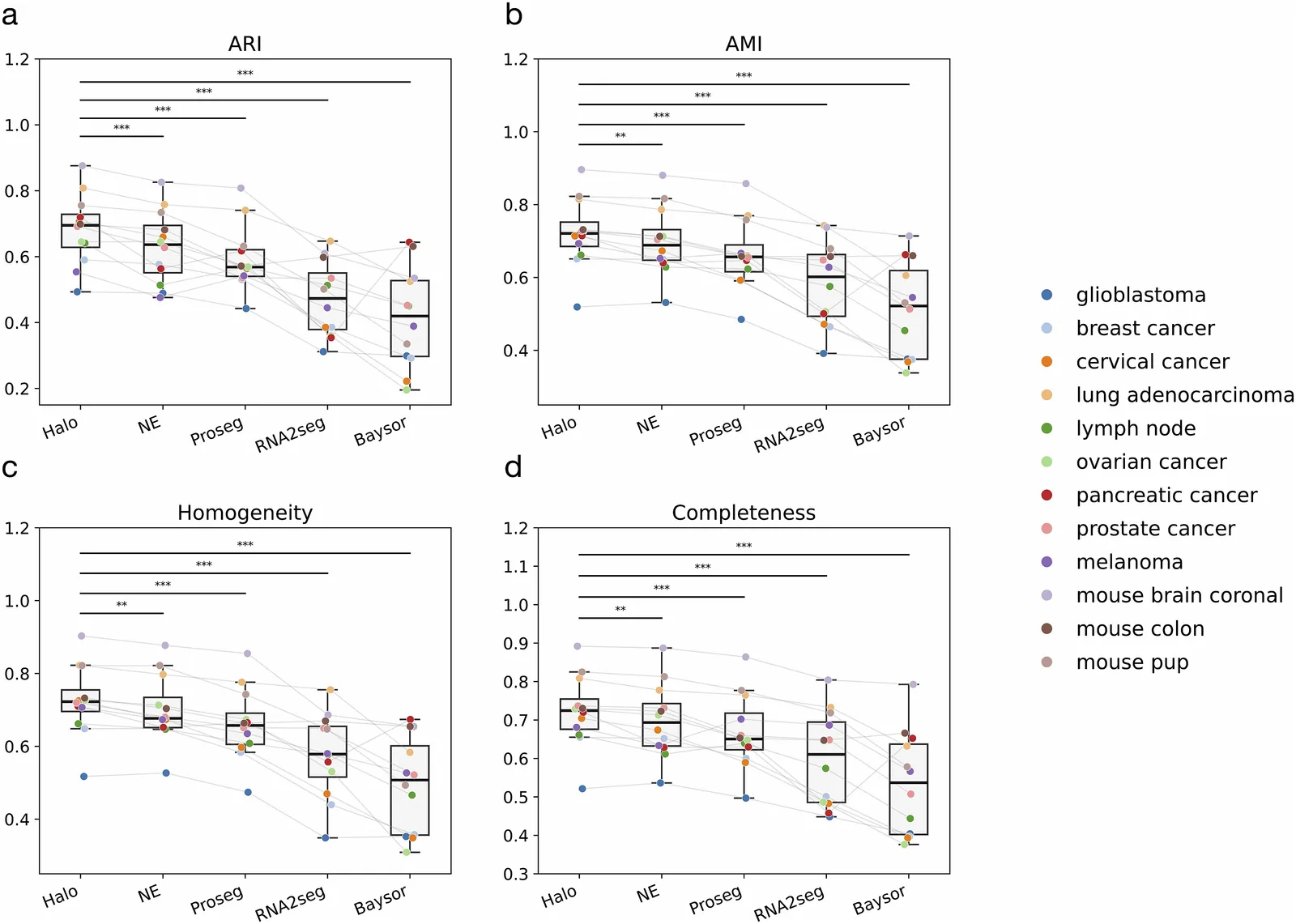
Evaluation of clustering performance for Halo, NE, Proseg, RNA2seg, and Baysor. (a) ARI, (b) AMI, (c) Homogeneity, and (d) Completeness. Each point represents the corresponding clustering agreement metric for the cells within that tissue. Statistical significance was assessed using a paired two-sided Wilcoxon signed-rank test. *** indicates *P*-value <.001; ** indicates *P*-value <.01.

We applied the same cell clustering pipeline to the gene expression count matrices derived from the ground truth whole-cell boundaries, as well as from the boundaries identified by Halo and competing methods. We quantitatively compared the cell clustering results derived from Halo and competing methods with those from the ground truth using metrics including the adjusted Rand index (ARI), adjusted mutual information (AMI), homogeneity, and completeness (Fig. 4). Halo achieves the best clustering metrics in nearly all tissues and shows significantly improved clustering performance overall across tissues. These results suggest that Halo enables more accurate cell clustering.

**Figure 5:**
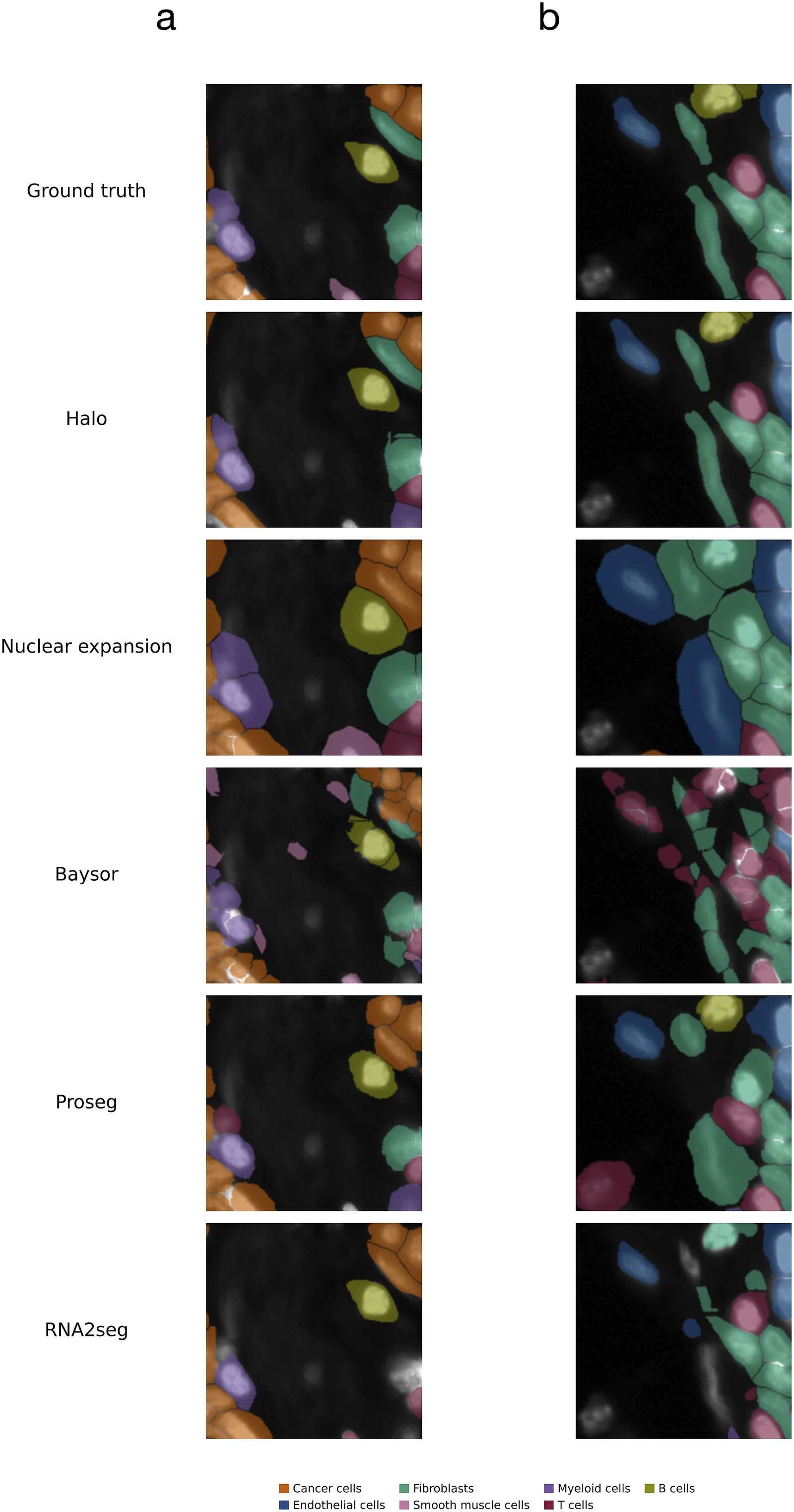
Examples of cell-type annotation based on the ground truth segmentation, Halo, NE, Baysor, Proseg, and RNA2seg, in human lung adenocarcinoma (a) and human melanoma (b).

We then performed cell-type annotation for the cell clusters identified using each segmentation method. Figure 5 shows the cell-type identification results produced by these methods in several example tissue regions. While the cell-type annotations based on Halo largely agree with those based on the ground truth, the annotations derived from other methods exhibit clear discrepancies in several cases. For example, NE, Proseg, and RNA2seg can mistakenly annotate fibroblasts as cancer cells, potentially biasing analyses of cancer–immune interactions.

### Halo more accurately captures cell morphological features

**Figure 6:**
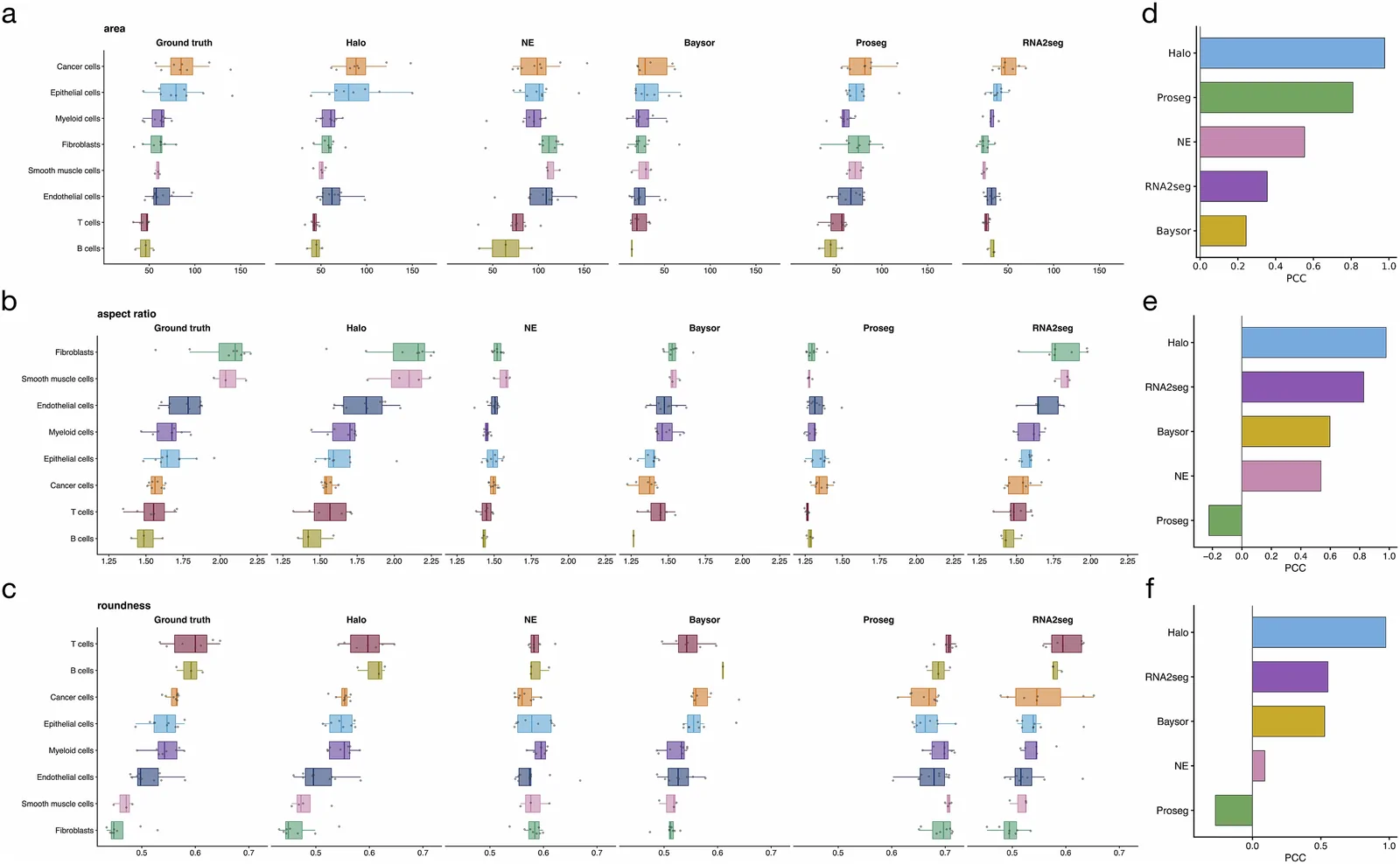
(a–c) Distributions of morphological features across cell types for different segmentation methods. (a) Cell area. (b) Aspect ratio. (c) Roundness. (d–f) Median Pearson correlation coefficients across datasets between the cell-type specific morphological feature vectors obtained using each segmentation method and the corresponding ground-truth vectors. (d) Cell area. (e) Aspect ratio. (f) Roundness.

By jointly profiling the morphological and transcriptomic features of the same cell, ST enables the study of cell morphology in a cell-type-specific manner, which is important for understanding cellular states, disease progression, and tissue organization [28–30]. To evaluate whether the whole-cell segmentation produced by Halo can be used for morphological analysis, we calculated several morphological features, including cell size, aspect ratio, and roundness, using ground-truth whole-cell boundaries as well as boundaries derived from Halo or competing methods (Fig. 6). The morphological features obtained using Halo are highly consistent with those from the ground truth and reflect known biological characteristics. For example, lymphocytes such as T cells and B cells exhibit relatively small areas and high roundness, reflecting their compact and spherical morphology, whereas fibroblasts and smooth muscle cells show larger aspect ratios and lower roundness due to their elongated, spindle-like shapes. Epithelial cells tend to have larger cell areas, consistent with their sheet-like organization in tissues. These patterns agree with well-established observations from quantitative cell morphology studies, indicating that the extracted features capture biologically meaningful differences between cell types [31–33]. In contrast, competing methods substantially distort morphological features and result in lower agreement with the ground truth (Fig. 6). For example, there is much less variation in aspect ratio and roundness across cell types for cell masks derived using NE, likely because the inferred whole-cell boundaries are circular or elliptical in most cases.

**Figure 7:**
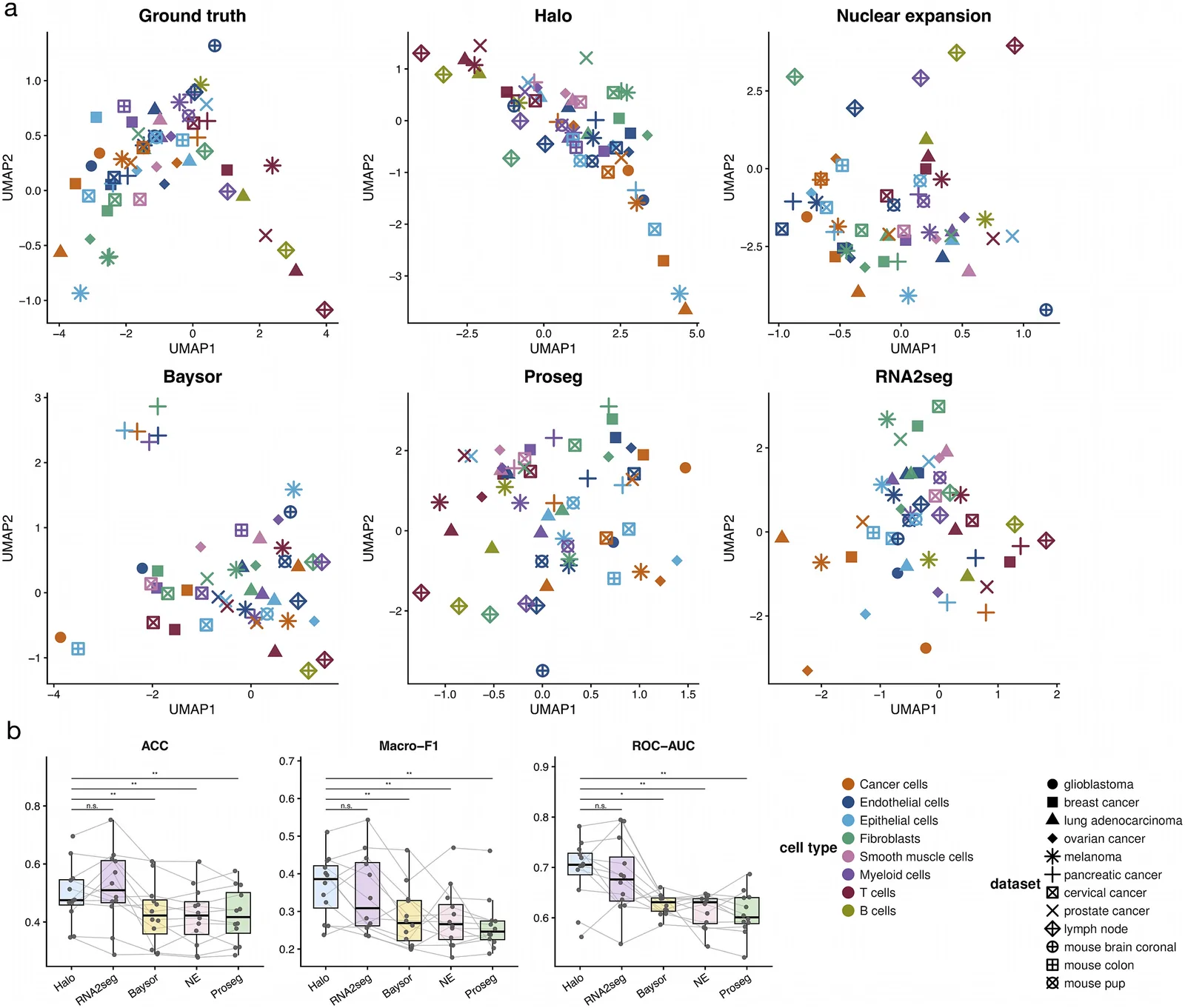
UMAP visualization and random forest analysis of morphological features. (a) Cell-type centroids across datasets in UMAP space. (b) Accuracy (left), macro-F1 (middle), and macro ROC–AUC (right) of the random forest classifier. Each point represents the mean classification performance for that dataset. Statistical significance was assessed using a paired two-sided Wilcoxon signed-rank test. ** indicates *P*-value <.01, * indicates *P*-value <.05, n.s. indicates *P*-value not significant.

Finally, we tested whether morphological features can be used to separate or predict different cell types. We first projected the morphological features calculated for each cell type within each tissue into a low-dimensional space using Uniform Manifold Approximation and Projection (UMAP) (Fig. 7a). Similar to the ground truth, morphological features derived from Halo lead to clear separation of different cell types, while the same cell types from different tissues remain close to one another. In contrast, when morphological features derived from NE are used, different cell types appear largely mixed in the UMAP embedding. We further evaluated cell-type prediction accuracy in a held-out test-set evaluation study using only morphological features as predictors. Halo achieves significantly higher prediction accuracy than competing methods in nearly all scenarios (Fig. 7b). Per-class cell numbers for each tissue and segmentation method are reported in Supplementary Table S1. These results suggest that morphological features derived from Halo more effectively separate and predict different cell types across tissues.

## Discussion

In this study, we introduce Halo, a pretrained model that enables accurate whole-cell segmentation using nuclei staining images and spatial locations of RNA transcripts as input. By converting RNA spatial locations into a pseudoimage, Halo effectively integrates these two data modalities and allows the use of existing segmentation methods developed for imaging data. We demonstrate that Halo substantially improves agreement with the 10*×* reference segmentation and downstream analyses compared to competing methods.

An important consideration in interpreting these results is that most quantitative evaluations in this study are referenced, directly or indirectly, to the 10*×* multimodal segmentation. Image IoU and Gene IoU directly quantify agreement with the reference cell masks and transcript assignments, while the clustering agreement and transcript-assignment analyses similarly depend on outputs derived from the same reference segmentation. In contrast, the incompatible-marker co-assignment score provides a reference-independent measure of biological plausibility. On this metric, Halo showed performance comparable with the competing methods, including NE, and the methods were not clearly distinguished. One possible explanation is that the incompatible-marker score measures a different and relatively coarse aspect of segmentation quality, namely the mixing of a selected set of biologically incompatible marker transcripts, and may be insensitive to differences in the precise placement of cell boundaries that nevertheless affect Image IoU and Gene IoU. Taken together, our results support higher agreement of Halo with the 10*×* multimodal reference segmentation, while the reference-independent analysis does not provide evidence of a corresponding advantage in incompatible-marker co-assignment.

While Halo currently relies on the Cellpose-SAM model, the training data can be directly used by other models that support cell or instance segmentation. Therefore, Halo can be readily improved as more powerful models pretrained on larger datasets become available in the future. To facilitate such developments, we have made the training data openly accessible.

When constructing pseudoimages from the spatial locations of RNA transcripts, Halo does not distinguish transcripts from different genes. Although transcripts from certain genes may preferentially localize to specific subcellular regions, incorporating such information would require the same gene set to be used during both training and inference on new datasets. Because different ST platforms and experiments often measure different gene panels, Halo does not differentiate transcripts by gene identity, which helps maximize generalizability across datasets with varying gene panels.

By default, Halo generates discrete, nonoverlapping cell masks and assigns each transcript to a single cell based on whether its spatial coordinate falls within the predicted boundary. The majority of the evaluations in this study were conducted using this standard hard-assignment framework. Halo additionally offers an optional diagnostic confidence score based on the Cellpose-SAM cellprob value, which reflects the pixel-level evidence that each transcript location lies within a cellular region. As shown in Supplementary Fig. S3, we compared transcript assignment accuracy across the confidence scores reported by Halo and Baysor. Transcript assignment accuracy increased gradually and consistently with the Halo confidence score across datasets. In contrast, Baysor did not show the same consistent increase, and in many datasets its assignment accuracy remained largely unchanged across the lower range of confidence scores. These results suggest that the Halo confidence score more effectively stratifies transcripts according to assignment reliability than the confidence score reported by Baysor. However, because the Halo confidence score is defined at the pixel level rather than for a specific transcript-to-cell relationship, it is not a calibrated posterior probability that a transcript belongs to a particular cell. Consequently, Halo’s hard, nonoverlapping masks cannot explicitly represent transcript-level assignment ambiguity between neighboring cells, particularly at cell-type boundaries.

Because the cell-type annotations in Fig. 5 are generated using marker gene-based annotation with the sctype package, and these labels are subsequently used in the cell morphology analyses, including the cell-type prediction analysis in Fig. 7b, these results may be influenced by the choice and specificity of the marker gene sets used for annotation.

## Methods

### Halo

#### Data collection and preprocessing

To train the Halo models, we collected 15 10*×* Genomics Xenium samples from the 10*×* Genomics website, spanning 12 distinct human and mouse tissue types. Detailed information for all samples is provided in Supplementary Table S2. For each sample, three types of information were collected: the DAPI nuclei staining image, whole-cell boundaries derived from multimodal staining, and the spatial locations of RNA transcripts. DAPI images were intensity-normalized by capping pixel values at the 99th percentile and then scaling all intensities to the range *[0,1]*. For the transcript data, only gene transcripts with a quality score (Q-score) *≥ 20* were retained, consistent with the default quality-control threshold used by the 10*×* Genomics Xenium processing pipeline. Halo also allows users to specify alternative Q-score cutoffs. In human breast cancer and human pancreatic cancer, varying the Q-score cutoff among 15, 20, and 30 had minimal effects on Image IoU and Gene IoU (Supplementary Fig. S4).

From each sample, we randomly selected 1000 image tiles, each containing at least 200 pixels within cellular regions and a minimum of 15 cells. Collectively, the selected tiles contained more than one million cells across all samples. The tiles from each sample were randomly partitioned into 800 training tiles and 200 test tiles. All training tiles were used to train Halo. For downstream performance evaluation, only the test tiles from 12 of the 15 samples were retained, as gene expression matrices for the remaining three samples could not be reliably retrieved due to technical issues with the 10*×* Xenium Ranger pipeline.

Throughout this study, we refer to the 10*×* multimodal whole-cell segmentation as the “ground truth” for evaluation purposes. This term denotes the reference segmentation used for quantitative comparisons rather than independently validated biological ground truth.

#### Transcript-density pseudo-image

Let $\{x_{i}\}_{i=1}^{N} \subset \mathbb{R}^{2}$ denote the spatial coordinates of the *N* detected RNA transcripts, where $x_{i} = \big (x_{i}^{(1)}, x_{i}^{(2)}\big )$ is the 2D location of transcript *i*.

We construct a transcript-density pseudo-image *f(u)* by placing a 2D Gaussian kernel at each transcript location and summing their contributions:


\begin{align*} & f(u) = \sum_{i=1}^{N} \exp\left( - \frac{\|u - x_{i}\|_{2}^{2}}{2\sigma^{2}} \right), \end{align*}

where $u \in \mathbb{R}^{2}$ denotes a spatial coordinate. We use *σ = 2.5* as the default bandwidth and for all main results reported in this study, as it achieved strong and stable performance across datasets while avoiding tissue-specific hyperparameter tuning. We evaluated *σ* values of 0.5, 1, 2.5, 5, and 10 separately within each tissue. As shown in Supplementary Fig. S5, both Image IoU and Gene IoU remained relatively stable across this range within individual tissues, with only modest variation across *σ* values. Users may optionally select alternative values of *σ*.

In addition to the Gaussian kernel, Halo supports exponential and uniform kernels as alternative options. The exponential kernel is defined as follows:


\begin{align*} & f_{\mathrm{E}}(u) = \sum_{i=1}^{N} \exp\left( -\frac{\|u-x_{i}\|_{2}}{\lambda} \right), \qquad \lambda=1.44. \end{align*}

The uniform kernel is defined as follows:


\begin{align*} & f_{\mathrm{U}}(u) = \sum_{i=1}^{N} \mathbb{I} \left( |u_{1}-x_{i1}|\leq c \ \mathrm{and}\ |u_{2}-x_{i2}|\leq c \right), \qquad c = 4 \end{align*}

Kernel parameters were selected by matching the radial second moment, $\mathbb{E}[r^{2}]$, to that of the Gaussian kernel. This yields comparable spatial spreads across kernels, allowing performance differences to primarily reflect kernel shape rather than effective spatial scale.

For the 2D exponential kernel, $\mathbb{E}_{K_{\mathrm{E}}}[r^{2}]=6\lambda ^{2}$. Matching this to the Gaussian second moment, $\mathbb{E}_{K_{\mathrm{G}}}[r^{2}]=2\sigma ^{2}$, gives $\lambda =\sigma /\sqrt{3}\approx 1.44$ for *σ =2.5*.

For a uniform square kernel over $[-c,c]^{2}$, $\mathbb{E}_{K_{\mathrm{U}}}[r^{2}]=2c^{2}/3$. Matching this to $\mathbb{E}_{K_{\mathrm{G}}}[r^{2}]=2\sigma ^{2}$ gives $c=\sqrt{3}\sigma \approx 4.33$ for *σ =2.5*. Because the half-width must be an integer on the discrete pixel grid, we used *c=4*.

To assess the effect of kernel choice on Halo performance, we trained additional Halo models using exponential and uniform kernels. All models were trained and evaluated using identical data splits, model architectures, and training settings. Performance was compared using Image IoU and Gene IoU. Halo showed only minor performance variation across the three kernel choices, as shown in Supplementary Fig. S6, indicating that the method is robust to the kernel used to construct the transcript pseudo-image. Therefore, users may select alternative kernels in practice while obtaining highly consistent results.

In practice, *f(u)* is evaluated on the discrete pixel grid of the image domain to obtain the transcript-density pseudo-image.

To ensure compatibility with the scaled DAPI image, the pixel values of the transcript-density pseudo-image were linearly scaled to the range *[0,1]*. The scaled DAPI image and the scaled transcript-density image were then concatenated to form a two-channel input image.

#### Model training

Cellpose-SAM (version 4.0.7) was trained to predict whole-cell boundaries from the two-channel input image. All training tiles across all training samples were included in the training process. The model was trained for 300 epochs using a learning rate of 0.001 and a weight decay of 1e-5.

#### Model inference

The trained Halo model can be applied to new ST datasets. For each dataset, a transcript-density pseudo-image must first be generated, and the corresponding two-channel input image constructed using the same procedure described above. The resulting two-channel images are then used as input to the trained Halo model for whole-cell boundary prediction. Halo additionally reports a diagnostic transcript confidence score, defined as the sigmoid-transformed Cellpose-SAM cellprob value at each transcript location. This pixel-level score reflects the evidence that a transcript lies within a cellular region, rather than the probability that it belongs to a specific cell.

### Competing method

All competing methods were applied to the same test datasets used to evaluate Halo, using the settings described below.

#### Nuclear expansion

The NE method was included as a baseline for comparison. Specifically, 10*×* Xenium Ranger was applied to all test datasets in this study using its built-in NE strategy, in which nuclear masks were uniformly expanded by 5*μ*m, unless the expansion encountered the boundary of a neighboring cell, to approximate whole-cell boundaries.

#### Baysor

Baysor (v0.7.1) was run using Xenium nucleus-based transcript assignments as prior segmentation, with min_molecules_per_cell = 20, prior_segmentation_confidence = 0.5, min_molecules_per_segment = 5, confidence:nn_id = 11, min_molecules_per_gene = 1, n_clusters = 4, iters = 500, force:2d = false, and unassigned_prior_label = “0”. The scale parameter was set to −1, with scale and scale_std automatically estimated from the prior segmentation separately for each dataset.

#### Proseg

Proseg (v3.1.1) was run using Xenium nucleus-based transcript assignments together with the corresponding nuclear segmentation masks, with cell_id_unassigned = 0, ignore_z_coord = true, cellpose_scale = 1, burnin_samples = 200, samples = 200, recorded_samples = 100, burnin_voxel_size = 2 *μ*m, voxel_size = 1 *μ*m, ncomponents = 10, nhidden = 100, hillclimb = 50, cell_compactness = 0.04, morphology_steps_per_iter = 4000, nuclear_reassignment_prob = 0.2, prior_seg_reassignment_prob = 0.5, diffusion_probability = 0.2, diffusion_sigma_near = 1, diffusion_sigma_far = 4, diffusion_sigma_z = 0.1, diffusion_proposal_sigma = 4, diffusion_proposal_sigma_z = 0.2, max_transcript_nucleus_distance = 60, quad_size = 150, burnin_dispersion = 1, ab_nihlo_bubble_prob = 0.05, nunfactored = 300, density_bandwidth = 0.5, and density_bins = 5. Transcript diffusion and gene-expression factorization were enabled, while connectivity enforcement, variable burn-in dispersion, and cell-specific scale factors were disabled.

#### RNA2seg

RNA2seg (v0.1.5) was run using the default_pretrained model with flow_threshold = 0.9, min_cell_size = 200, cellbound_flow_threshold = 0.4, and min_area = 0.

### Performance evaluations: intersection over union and transcript assignment accuracy

#### Image intersection over union

The image-level IoU was computed separately within each test image tile. Both the ground-truth and predicted whole-cell boundaries were represented as labeled images, where background pixels were assigned a value of *0* and each individual cell was assigned a unique positive integer label.

Let $G_{i}$ denotes the pixel set corresponding to ground-truth cell *i*, and let $P_{j}$ denotes the pixel set corresponding to predicted cell *j*. The IoU between $G_{i}$ and $P_{j}$ was defined as


\begin{align*} & \mathrm{IoU}(i, j) = \frac{|G_{i} \cap P_{j}|}{|G_{i} \cup P_{j}|}, \end{align*}

where *|· |* denotes the number of pixels in the set. This definition produces an IoU matrix whose rows correspond to ground-truth cells and whose columns correspond to predicted cells.

For each predicted cell *j*, its IoU score was defined as


\begin{align*} & \mathrm{IoU}_{j} = \max_{i} \, \mathrm{IoU}(i, j). \end{align*}

The final image-level IoU was computed as the mean of $\mathrm{IoU}_{j}$ across all predicted cells, and results were averaged across tiles.

#### Gene intersection over union

For each cell, transcripts were assigned based on spatial containment within the corresponding segmentation mask. Only transcripts that passed quality control filtering were included in the analysis.

For a given ground-truth cell *i* and predicted cell *j*, let $\mathscr{K}_{i}$ and $\mathscr{L}_{j}$ denote the sets of transcripts assigned to each cell, respectively. The gene-level IoU was defined as


\begin{align*} & \mathrm{GeneIoU}(i, j) = \frac{|\mathscr{K}_{i} \cap \mathscr{L}_{j}|} {|\mathscr{K}_{i} \cup \mathscr{L}_{j}|}. \end{align*}

For each predicted cell *j*, its gene IoU score was defined as


\begin{align*} & \mathrm{GeneIoU}_{j} = \max_{i} \, \mathrm{GeneIoU}(i, j). \end{align*}

Gene IoU scores were averaged across predicted cells within each tile and subsequently averaged across tiles to obtain dataset-level summary statistics.

#### Incompatible-marker co-assignment score

For each cell type, we curated a set of marker genes that are highly expressed specifically in that cell type (Supplementary Table S3). Cells with fewer than 10 assigned transcripts or no transcripts corresponding to any curated marker gene were excluded. For each remaining cell, we counted the transcripts assigned to the marker-gene set of each cell type. The dominant cell type was defined as the cell type whose marker genes accounted for the largest number of assigned transcripts. Transcripts corresponding to marker genes of all non-dominant cell types were classified as incompatible markers. The incompatible-marker co-assignment score for each cell was defined as the proportion of all assigned transcripts that were classified as incompatible markers. For each sample, the score was averaged across all included cells.

#### Transcript assignment accuracy stratified by distance to the cell centroid

Each predicted cell mask was first matched to the ground-truth cell mask with which it had the highest image IoU. All detected transcripts, including those left unassigned by the ground-truth segmentation, were included. A transcript was considered correctly assigned if its predicted cell assignment corresponded to the same cell identified by the ground-truth segmentation, or if it remained unassigned in both the predicted and ground-truth segmentations. All other outcomes were classified as incorrect. For transcripts assigned by the ground-truth segmentation, distance was measured from the transcript to the centroid of its ground-truth cell. For ground-truth-unassigned transcripts, distance was measured to the centroid of the nearest ground-truth cell. Assignment accuracy was calculated within three distance intervals: 0–5 *μ*m, 5–10 *μ*m, and *≥ 10*  *μ*m.

### Performance evaluation: cell clustering by gene expression

#### Cell alignment

To facilitate comparison of cells obtained from different methods, the cell boundary masks generated by Halo or by the competing methods were aligned to the ground-truth cell boundary masks. Specifically, a one-to-one correspondence was established between a cell *i*, whose mask was obtained from Halo, and a cell *j* in the ground truth if the following three criteria were satisfied: (i) the cell-level IoU between *i* and *j*, calculated as described above, was at least 0.3; (ii) among all cells identified by Halo, cell *i* had the highest IoU with cell *j*; and (iii) among all ground-truth cells, cell *j* had the highest IoU with cell *i*.

The same procedure was applied to establish cell alignment between cells identified by the competing methods and the ground truth.

#### Cell clustering

For the ground-truth cell boundaries and those generated by Halo or the competing methods, the corresponding cell-by-gene count matrices were computed using 10*×* Xenium Ranger. Only cells successfully aligned in the preceding step were retained for downstream analysis. Each count matrix was then processed using the Python package Scanpy. Briefly, the data were normalized by library size and log-transformed, followed by principal component analysis (PCA). A *k*-nearest-neighbor graph was constructed in the PCA space, and Leiden clustering was performed with the resolution parameter fixed at 1.

#### Cell-type annotation

Cell-type annotation was conducted for each cluster using the sctype R package, based on curated cell-type specific marker genes. For each cancer dataset, a cancer specific marker gene set was additionally applied to identify malignant cell populations. The full list of cell types and their corresponding marker genes used in this study is provided in Supplementary Table S4.

#### Cluster agreement

Agreement between clustering results from Halo or the competing methods and the ground-truth reference was evaluated on aligned cells. Concordance was quantified using the ARI, AMI, homogeneity, and completeness, computed with adjusted_rand_score, adjusted_mutual_info_score, homogeneity_score, and completeness_score from the scikit-learn library.

### Performance evaluation: cell morphology

#### Morphological features

For each segmented cell, a set of morphological features was computed from its corresponding cell boundary mask.

**Area.** Cell area was defined as the area enclosed by the segmented cell boundary polygon.

**Perimeter.** Cell perimeter was computed as the total length of the segmented polygon boundary.

**Feret’s diameter.** Feret’s diameter was defined as the maximum pairwise Euclidean distance between any two points on the convex hull of the cell polygon.

**Eccentricity.** Cell eccentricity was quantified using PCA of the polygon vertex coordinates. It was defined as $1 - \lambda _{2} / \lambda _{1}$, where $\lambda _{1}$ and $\lambda _{2}$ denote the variances explained by the first and second principal components, respectively. Values closer to 1 indicate more elongated shapes, whereas values closer to 0 indicate more isotropic shapes.

**Roundness.** Roundness was defined as the ratio between the cell area and the area of its minimum bounding circle. Values approaching 1 indicate shapes that closely approximate a circle and efficiently fill their bounding circle, whereas smaller values reflect elongated or irregular morphologies.

**Circularity.** Circularity was computed as $4 \pi A / P^{2}$, where *A* denotes the cell area and *P* denotes the cell perimeter. A perfect circle yields a circularity of 1, whereas lower values indicate increasingly irregular or elongated boundaries.

**Solidity.** Solidity was defined as the ratio between the cell area and the area of its convex hull. This metric quantifies boundary concavity, with values near 1 indicating nearly convex shapes and lower values indicating the presence of indentations or fragmented outlines.

**Aspect ratio.** Aspect ratio was computed from the minimum-area rotated bounding rectangle of each cell as the ratio between the rectangle width and height. Larger values correspond to more elongated shapes, whereas values near 1 indicate more equiaxed cell geometries.

#### Uniform Manifold Approximation and Projection dimension reduction

All morphological features described above were used as input. Each feature was standardized across cells using z-score transformation. UMAP was then performed using the uwot package (version 0.2.3), with n_neighbors = 30, min_dist = 0.01, and Euclidean distance.

To visualize the global organization of cell morphologies, tissue-level centroids were computed by averaging embedding coordinates of cells within each dataset–cell type combination. These centroids were then plotted, with colors indicating cell types and point shapes representing datasets.

#### Random forest classification

To evaluate whether cell morphological features could discriminate cell types, we trained a random forest classifier separately for each tissue. For each tissue, cells labeled as unknown or unassigned were excluded before model fitting. Morphological features were extracted from the selected feature columns and only numeric features were retained for modeling.

For each tissue-specific dataset, the samples were divided into training and test sets using a stratified split, such that each cell type was represented in both sets while *∼*20% of cells were assigned to the test set. Cell-type labels were treated as the response variable, and the morphological features were used as predictors. Random forest models were trained using the “randomForest” R package with class weighting enabled, where class weights were set inversely proportional to class frequency in the training set to partially mitigate class imbalance. Unless otherwise specified, models were trained with 500 trees for tissue-level analyses.

Model performance was evaluated on the held-out test set using three complementary metrics: accuracy, macro-F1, and macro ROC–AUC.

Accuracy was defined as the proportion of correctly classified cells among all cells in the test set:


\begin{align*} & \mathrm{Accuracy} = \frac{\text{number of correct predictions}}{\text{total number of predictions}}. \end{align*}

This metric summarizes overall classification performance but may be influenced by class imbalance.

Macro-F1 was used to assess balanced multiclass performance across cell types. For each class, precision and recall were calculated in a one-vs-rest manner:


\begin{align*} & \mathrm{Precision}_k = \frac{{\mathrm{TP}}_k}{{\mathrm{TP}}_k + {\mathrm{FP}}_k}, \qquad \mathrm{Recall}_k = \frac{{\mathrm{TP}}_k}{{\mathrm{TP}}_k + {\mathrm{FN}}_k}, \end{align*}

where ${\mathrm{TP}}_{k}$, ${\mathrm{FP}}_{k}$, and ${\mathrm{FN}}_{k}$ denote the true positives, false positives, and false negatives for class *k*, respectively. The class-specific F1 score was then computed as


\begin{align*} & F1_k = \frac{2 \cdot \mathrm{Precision}_k \cdot \mathrm{Recall}_k}{\mathrm{Precision}_k + \mathrm{Recall}_k}. \end{align*}

Macro-F1 was obtained by averaging the F1 scores across all classes:


\begin{align*} & \mathrm{Macro\mbox{-}F1} = \frac{1}{K} \sum_{k=1}^{K} F1_k, \end{align*}

where *K* is the number of classes. This metric gives equal weight to each class and is therefore more sensitive to minority-class performance than accuracy.

Macro ROC–AUC was used to quantify the probability-based discrimination ability of the classifier. For each class, a one-vs-rest receiver operating characteristic (ROC) curve was constructed from the predicted class probabilities, and the corresponding area under the curve (AUC) was computed. The final macro ROC–AUC was defined as the average of the class-specific AUC values:


\begin{align*} & \mathrm{Macro\ ROC\mbox{--}AUC} = \frac{1}{K} \sum_{k=1}^{K} \mathrm{AUC}_k. \end{align*}

These metrics reflect how well the model separates each cell type from the others.

The same procedure was applied to cell masks obtained from the competing methods and from the ground truth.

**Key Points**

- Accurate whole-cell segmentation is critical for reconstructing single-cell transcriptomes from spatial data, but remains challenging when only nuclear staining is available.
- Halo provides a pretrained and generalizable solution by integrating nuclear morphology with RNA spatial information, allowing direct application to new datasets without additional training.
- Improved segmentation enables more accurate RNA-to-cell assignment, enhances cell-type identification, and yields more reliable estimation of cell morphological features.

## Supplementary Material

Supplementary_material_bbag515

## Data Availability

All data used in this study were downloaded from the 10*×* Genomics website (https://www.10xgenomics.com/datasets). Detailed dataset locations are provided in Supplementary Table S2. The Halo software package and the finetuned model weights are freely available at https://huggingface.co/XYZ1998/Halo, and the training data are publicly available at https://zenodo.org/records/19201256.
