# Supplementary material for: Halo: a pretrained model for whole-cell segmentation from nuclei images in spatial transcriptomics
Source: Brief Bioinform. 2026 Sep 17;27(5):bbag515. doi: 10.1093/bib/bbag515 (PMC13584070; doi:10.1093/bib/bbag515)
Supplement: Supplementary file 1 [file supplementary_material_bbag515.zip › supp_bbag515_revise.pdf]

Supplementary materials

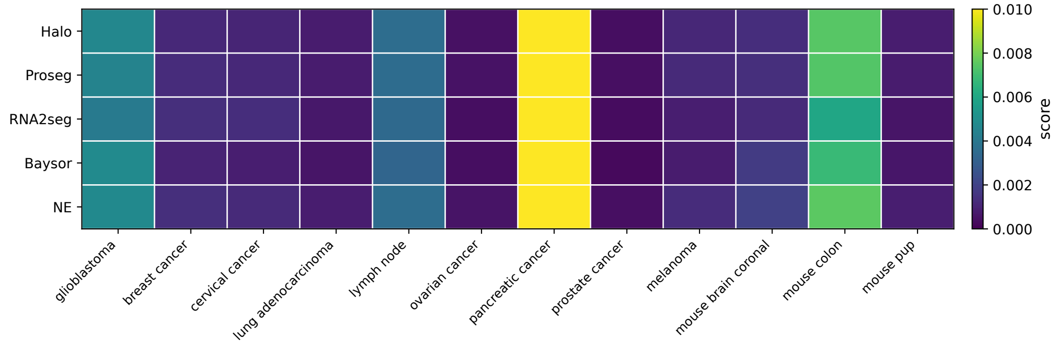

Figure S1. Incompatible-marker co-assignment scores averaged across 12 datasets.

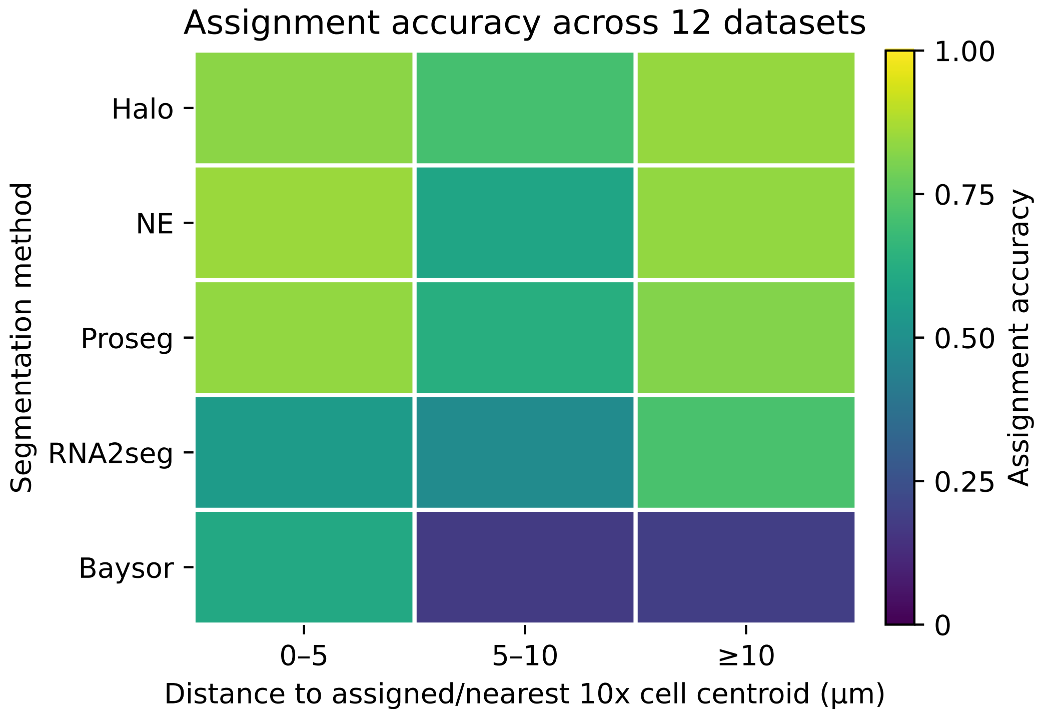

Figure S2. Transcript assignment accuracy averaged across 12 datasets for distances to the nearest 10x cell centroid of 0 to 5 μm, 5 to 10 μm, and greater than 10 μm.

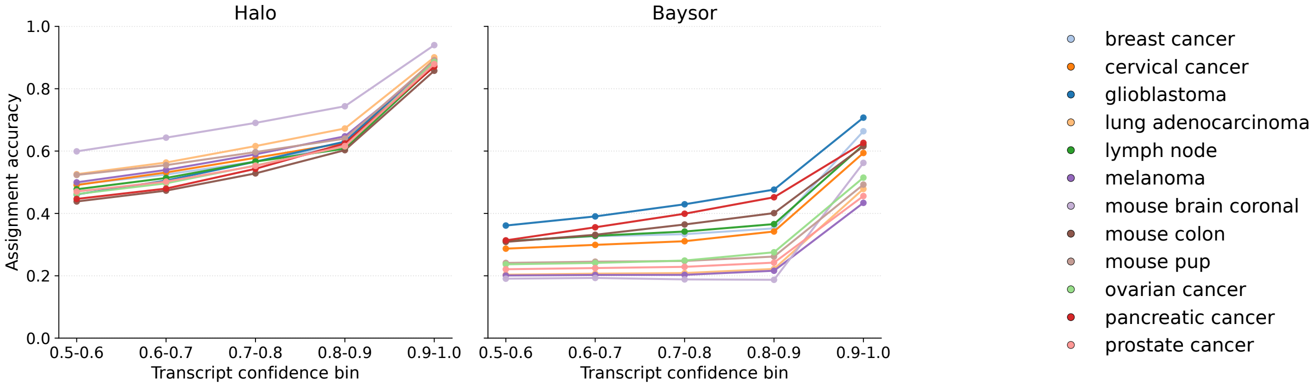

Figure S3. Association between confidence scores and assignment accuracy. Transcripts were grouped into confidence-score bins, and assignment accuracy was calculated for each bin.

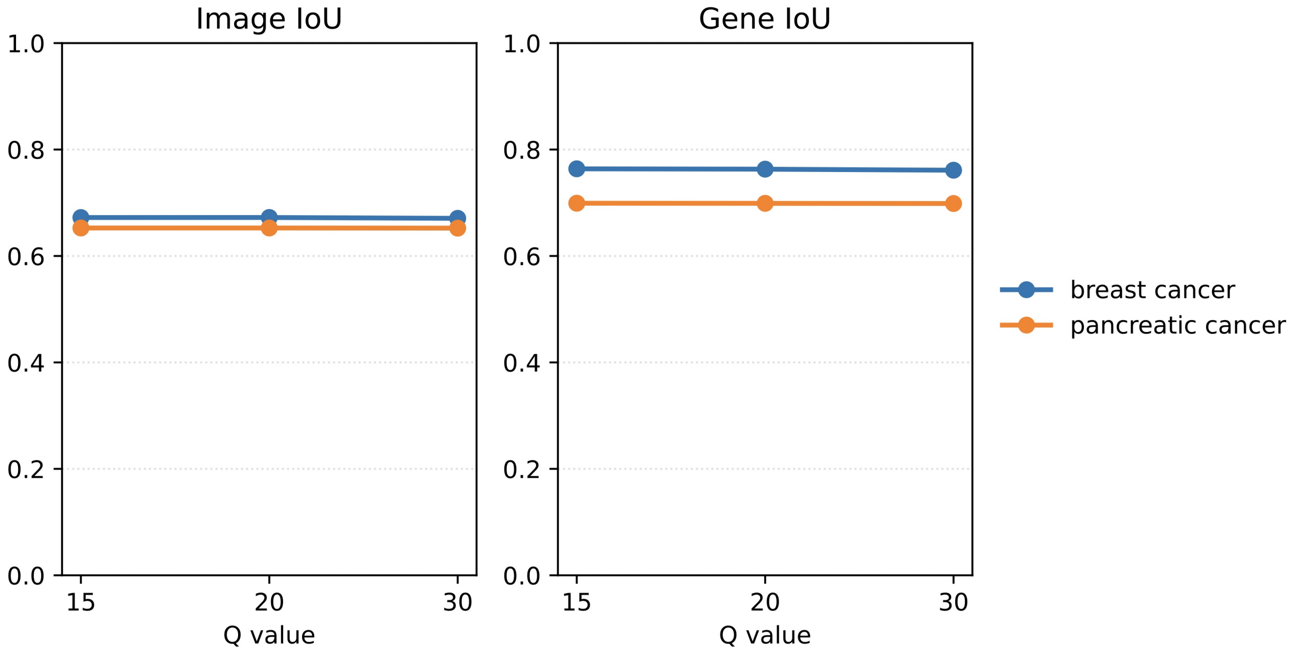

Figure S4. Image IoU and Gene IoU for human breast cancer and human pancreatic cancer across different Q-score thresholds used for transcript filtering.

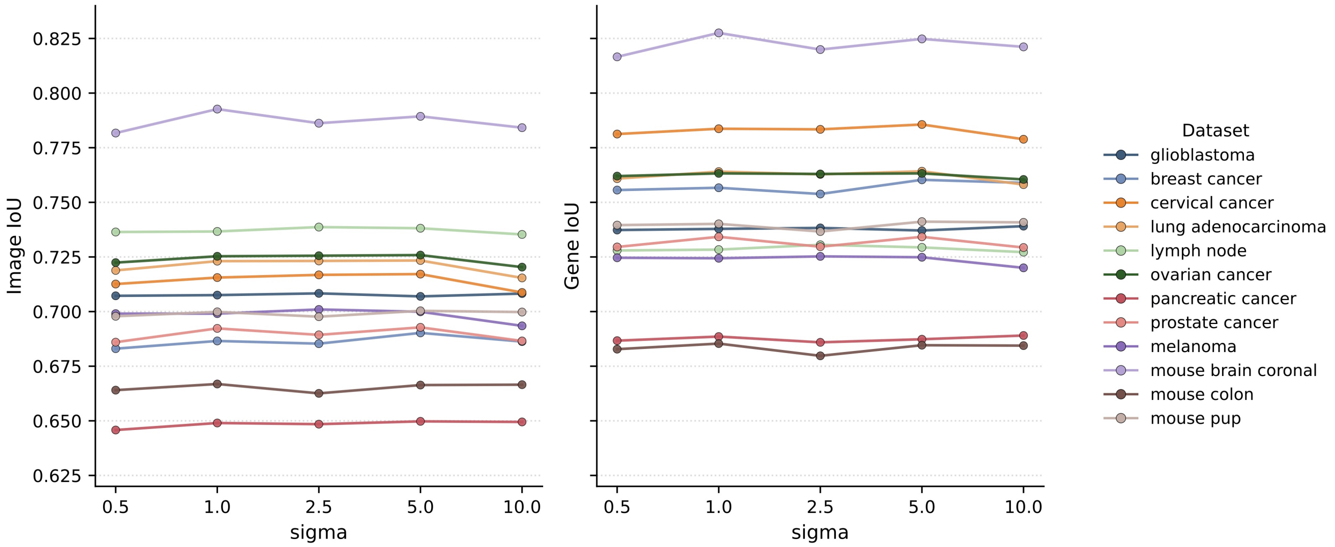

Figure S5. Sensitivity of Halo segmentation performance to Gaussian kernel bandwidth. Halo models were independently trained and evaluated using transcript pseudo-images constructed with σ values of 0.5, 1, 2.5, 5, and 10. Image IoU (left) and Gene IoU (right) are shown separately for each tissue, with each line representing an individual tissue.

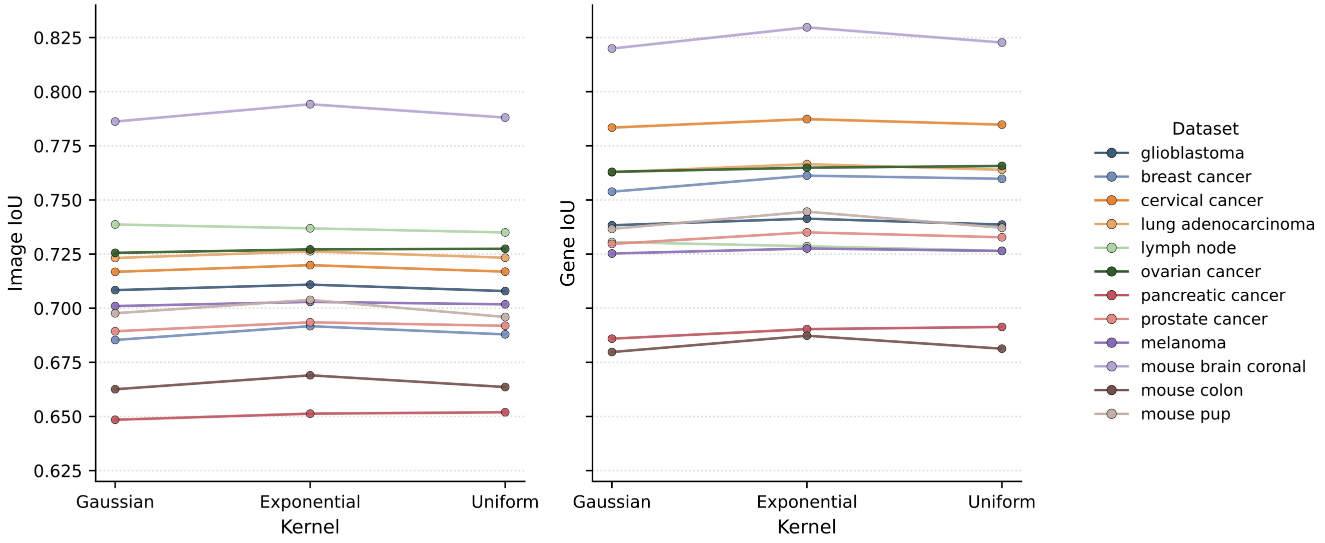

Figure S6. Image IoU (left) and Gene IoU (right) obtained using Gaussian, exponential, and uniform kernels to construct transcript pseudo-images for Halo.
